# Dicobalt copper bis­[orthophosphate(V)] monohydrate, Co_2.39_Cu_0.61_(PO_4_)_2_·H_2_O

**DOI:** 10.1107/S1600536810015382

**Published:** 2010-04-30

**Authors:** Abderrazzak Assani, Mohamed Saadi, Lahcen El Ammari

**Affiliations:** a Laboratoire de Chimie du Solide Appliquée, Faculté des Sciences, Université Mohammed V-Agdal, Avenue Ibn Battouta, BP 1014, Rabat, Morocco

## Abstract

In an attempt to hydro­thermally synthesize a phase with composition Co_2_Cu(PO_4_)_2_·H_2_O, we obtained the title compound, Co_2.39_Cu_0.61_(PO_4_)_2_·H_2_O instead. Chemical analysis confirmed the presence of copper in the crystal. The crystal structure of the title compound can be described as a three- dimensional network constructed from the stacking of two types of layers extending parallel to (010). These layers are made up from more or less deformed polyhedra: CoO_6_ octa­hedra, (Cu/Co)O_5_ square pyramids and PO_4_ tetra­hedra. The first layer is formed by pairs of edge-sharing (Cu/Co)O_5_ square pyramids linked *via* a common edge of each end of the (Cu/Co)_2_O_8_ dimer to PO_4_ tetra­hedra. The second layer is undulating and is built up from edge-sharing CoO_6_ octa­hedra. The linkage between the two layers is accomplished by PO_4_ tetra­hedra. The presence of water mol­ecules in the CoO_4_(H_2_O)_2_ octa­hedron also contributes to the cohesion of the layers through O—H⋯O hydrogen bonding.

## Related literature

For the properties of and background to metal phosphates, see: Clearfield (1988 ▶); Gao & Gao (2005 ▶); Viter & Nagornyi (2009 ▶); Harrison *et al.* (1995 ▶). For compounds with the same structure, see: Anderson *et al.* (1976 ▶); Liao *et al.* (1995 ▶); Sørensen *et al.* (2004 ▶); Moore & Araki (1975 ▶).

## Experimental

### 

#### Crystal data

Co_2.39_Cu_0.61_(PO_4_)_2_·H_2_O
*M* *_r_* = 387.57
Monoclinic, 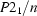

*a* = 8.086 (2) Å
*b* = 9.826 (3) Å
*c* = 9.042 (3) Å
β = 114.621 (1)°
*V* = 653.1 (3) Å^3^
*Z* = 4
Mo *K*α radiation
μ = 8.49 mm^−1^
*T* = 296 K
0.24 × 0.12 × 0.06 mm

#### Data collection

Bruker X8 APEX diffractometer
Absorption correction: multi-scan (*SADABS*; Bruker, 2005 ▶) *T*_min_ = 0.306, *T*_max_ = 0.601
13678 measured reflections
3531 independent reflections
3278 reflections with *I* > 2σ(*I*)
*R*_int_ = 0.023

#### Refinement

*R*[*F* ^2^ > 2σ(*F* ^2^)] = 0.022
*wR*(*F* ^2^) = 0.051
*S* = 1.10
3531 reflections
129 parameters
H-atom parameters constrained
Δρ_max_ = 1.01 e Å^−3^
Δρ_min_ = −0.79 e Å^−3^

### 

Data collection: *APEX2* (Bruker, 2005 ▶); cell refinement: *SAINT* (Bruker, 2005 ▶); data reduction: *SAINT*; program(s) used to solve structure: *SHELXS97* (Sheldrick, 2008 ▶); program(s) used to refine structure: *SHELXL97* (Sheldrick, 2008 ▶); molecular graphics: *ORTEP-3 for Windows* (Farrugia, 1997 ▶) and *DIAMOND* (Brandenburg, 2006 ▶); software used to prepare material for publication: *WinGX* (Farrugia, 1999 ▶).

## Supplementary Material

Crystal structure: contains datablocks I, global. DOI: 10.1107/S1600536810015382/wm2323sup1.cif

Structure factors: contains datablocks I. DOI: 10.1107/S1600536810015382/wm2323Isup2.hkl

Additional supplementary materials:  crystallographic information; 3D view; checkCIF report

## Figures and Tables

**Table 1:** Hydrogen-bond geometry (Å, °)

*D*—H⋯*A*	*D*—H	H⋯*A*	*D*⋯*A*	*D*—H⋯*A*
O9—H9*A*⋯O1	0.83	1.97	2.768 (2)	159
O9—H9*B*⋯O5^i^	0.92	2.27	2.942 (2)	130
O9—H9*B*⋯O4^ii^	0.92	2.30	2.905 (2)	123

Symmetry codes: (i) 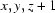; (ii) 
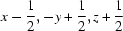
.

**Fig. 1.**
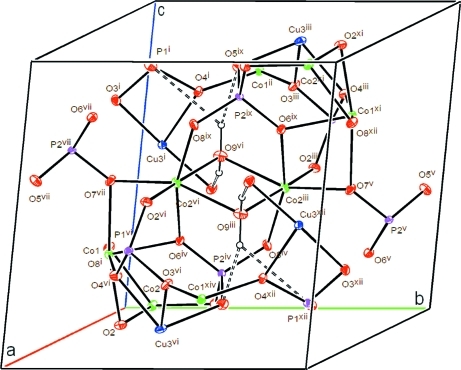
A three-dimensional view of the crystal structure of the Co2.39Cu0.61(PO4)2.H2O compound drawn with displacement parameters at the 60% probability level. H atoms are given as small spheres of arbitrary radius. For symmetry operators, see geometric parameters Table.

**Fig. 2.**
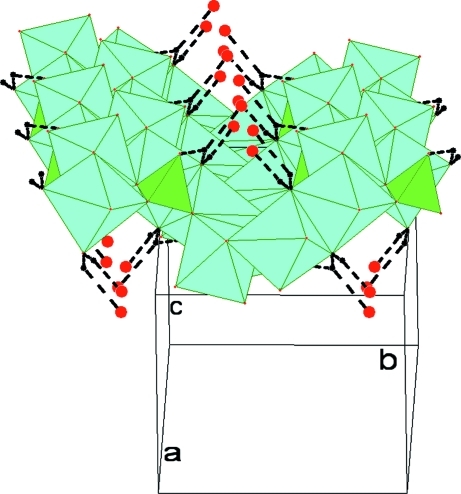
The undulated layer built up from edge sharing CoO6 octahedra and water molecules.

**Fig. 3.**
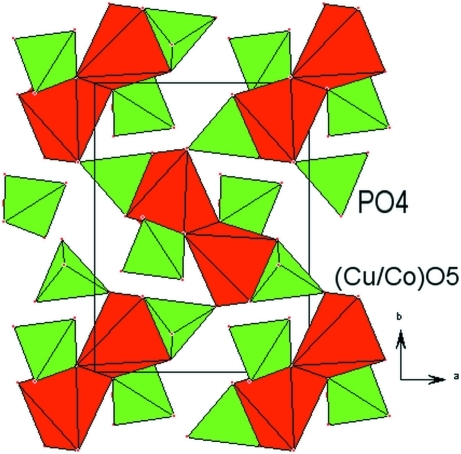
A copper phosphate layer formed by (Cu/Co)2P2O12 linked to PO4 tetrahedra.

**Fig. 4.**
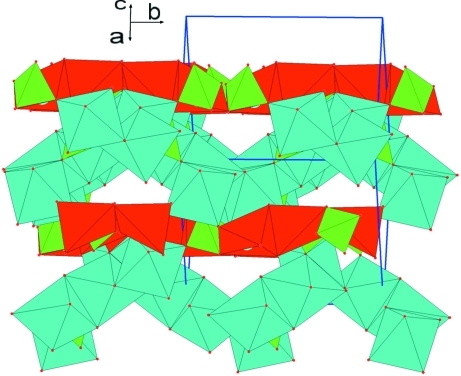
Stacking of the two types of layers projected approximately along [101].

## supplementary crystallographic information

### Comment

Metal-phosphates have received great attention owing to their applications such as catalysts (Viter & Nagornyi, 2009; Gao & Gao, 2005) and as ion-exchangers (Clearfield, 1988)). Mainly, the flexibility of the metal coordination and the possibility to generate anionic frameworks *M*^II^PO_4_^-^, analogous to AlSiO_4_^-^ in the well known aluminosilicate zeolites, offer a rich structural diversity of this family of compounds.

Our interest is particularly focused on the hydrothermally synthesized orthophosphates with formula *MM'*_2_(PO_4_)_2_.H_2_O (*M* and *M'*= bivalent cations). In this work, a new dicobalt copper bis[orthophosphate] monohydrate, Co_2.39_Cu_0.61_(PO_4_)_2_.H_2_O was synthesized and structurally characterized.

A three-dimensional view of the crystal structure of the title compound is given in Fig. 1. It shows that the metal cations are located in three crystallographically different sites, two octahedra entirely occupied by cobalt and one square-pyramid statistically filled with Co/Cu. Refinement of the occupancy of this metal site has led to the following composition, Co_2.39_Cu_0.61_(PO_4_)_2_.H_2_O. The cationic distributions indicate that Cu prefers the site with a lower coordination number, whereas Co prefers coordination number of 6. This may be attributed to a gain in crystal field stabilisation energy for Co^2+^ in the octahedral sites and to the small difference between the sizes of the two cations Co^2+^ and Cu^2+^.

The network is built up from three different types of polyhedra more or less distorted: CoO_6_ octahedra , (Cu/Co)O_5_ square-pyramids and PO_4_ tetrahedra. One octahedron (Co1), slightly distorted, has a coordination sphere composed of O atoms from PO_4_ groups, while that of the other (Co2) is made up of four O atoms from PO_4_ groups and by two water molecules (O9). This fact explains its more pronounced distortion, with Co—O bond lengths in the range 2.0407 (11)-2.3310 (13) Å.

All CoO_6_ octahedra are linked together by edge-sharing and sharing three corners of PO_4_ tetrahedra, in the way to built a layer parallel to (010) as shown in Fig. 2. Therefore, the presence of the water molecule involved in the formation of the CoO_4_(H_2_O)_2_ octahedron causes a corrugation in this layer through O—H···O hydrogen bonds. Furthermore, Fig. 3 shows that each pair of distorted square-pyramids share an edge and built up a dimer linked to two regular PO_4_ tetrahedra via a common edge. The sequence of (Cu/Co)O_5_ and PO_4_ polyhedra leads to the formation of another layer (Fig. 3). As a matter of fact, the network of this structure can be described by stacking these two types of layers as represented in Fig. 4.

Compounds isotypic with the title phase are relatively rare, however, there are four known compounds which adopt this structure, viz. Co_3_(PO_4_)_2_.H_2_O (Anderson *et al.*, 1976), CuMn_2_(PO_4_)_2_.H_2_O (Liao *et al.*, 1995), Co_2.59_Zn_0.41_(PO_4_)_2_.H_2_O (Sørensen *et al.*, 2004) and Fe_3_(PO_4_)_2_.H_2_O (Moore & Araki, 1975).

### Experimental

The crystals of the title compound were hydrothermally synthesized starting from a mixture of metallic copper (0.0381 g), basic cobalt(II) carbonate (0.0318 g), 85 %_wt_ phosphoric acid (0.10 ml) and 10 ml distilled water. The hydrothermal synthesis was carried out in 23 ml Teflon-lined autoclave under autogeneous pressure at 468 K during 24 h. The product was filtered off, washed with deionized water and air dried. The reaction product consists of two types of crystals. The first one, dark violet crystals, corresponds to the title compound with the refined composition Co_2.39_Cu_0.61_(PO_4_)_2_.H_2_O. An elemental chemical analysis (EDS) confirms the presence of copper in the crystal. The second type of crystals is identified to be the known cobalt hydroxy phosphate Co_2_(OH)PO_4_ (Harrison *et al.*, 1995).

### Refinement

All H atoms were initially located in a difference map and refined with a O—H distance restraint of 0.84 (1) Å. Later they were refined in the riding model approximation with U_iso_(H) set to 1.5 U_eq_(O). Refinements of the site ocupancy factors of the metal sites revealed the octahedrally coordinated sites solely occupied by Co, whereas the 5-coordinated site shows a mixed occupancy of Co:Cu = 0.387 (11):0.613 (11).

### Crystal data

**Table:**

Co_2.39_Cu_0.61_(PO_4_)_2_·H_2_O	*F*(000) = 745
*M**_r_* = 387.57	*D*_x_ = 3.942 Mg m^−^^3^
Monoclinic, *P*2_1_/*n*	Mo *K*α radiation, λ = 0.71073 Å
Hall symbol: -P 2yn	Cell parameters from 13678 reflections
*a* = 8.086 (2) Å	θ = 2.9–38.0°
*b* = 9.826 (3) Å	µ = 8.49 mm^−^^1^
*c* = 9.042 (3) Å	*T* = 296 K
β = 114.621 (1)°	Block, dark violet
*V* = 653.1 (3) Å^3^	0.24 × 0.12 × 0.06 mm
*Z* = 4	

### Data collection

**Table:**

Bruker X8 APEX diffractometer	3531 independent reflections
Radiation source: fine-focus sealed tube	3278 reflections with *I* > 2σ(*I*)
graphite	*R*_int_ = 0.023
φ and ω scans	θ_max_ = 38.0°, θ_min_ = 2.9°
Absorption correction: multi-scan (*SADABS*; Bruker, 2005)	*h* = −11→14
*T*_min_ = 0.306, *T*_max_ = 0.601	*k* = −16→16
13678 measured reflections	*l* = −15→15

### Refinement

**Table:**

Refinement on *F*^2^	Secondary atom site location: difference Fourier map
Least-squares matrix: full	Hydrogen site location: inferred from neighbouring sites
*R*[*F*^2^ > 2σ(*F*^2^)] = 0.022	H-atom parameters constrained
*wR*(*F*^2^) = 0.051	*w* = 1/[σ^2^(*F*_o_^2^) + (0.0196*P*)^2^ + 0.5902*P*] where *P* = (*F*_o_^2^ + 2*F*_c_^2^)/3
*S* = 1.10	(Δ/σ)_max_ = 0.001
3531 reflections	Δρ_max_ = 1.01 e Å^−^^3^
129 parameters	Δρ_min_ = −0.79 e Å^−^^3^
0 restraints	Extinction correction: *SHELXL97* (Sheldrick, 2008), Fc^*^=kFc[1+0.001xFc^2^λ^3^/sin(2θ)]^-1/4^
Primary atom site location: structure-invariant direct methods	Extinction coefficient: 0.0034 (3)

### Special details

**Table:**

Geometry. All esds (except the esd in the dihedral angle between two l.s. planes) are estimated using the full covariance matrix. The cell esds are taken into account individually in the estimation of esds in distances, angles and torsion angles; correlations between esds in cell parameters are only used when they are defined by crystal symmetry. An approximate (isotropic) treatment of cell esds is used for estimating esds involving l.s. planes.
Refinement. Refinement of *F*^2^ against ALL reflections. The weighted *R*-factor wR and goodness of fit *S* are based on *F*^2^, conventional *R*-factors *R* are based on *F*, with *F* set to zero for negative *F*^2^. The threshold expression of *F*^2^ > σ(*F*^2^) is used only for calculating *R*-factors(gt) etc. and is not relevant to the choice of reflections for refinement. *R*-factors based on *F*^2^ are statistically about twice as large as those based on *F*, and *R*- factors based on ALL data will be even larger.

### Fractional atomic coordinates and isotropic or equivalent isotropic displacement parameters (Å^2^)

**Table:**

	*x*	*y*	*z*	*U*_iso_*/*U*_eq_	Occ. (<1)
Cu3	0.14535 (2)	0.125674 (19)	−0.43994 (2)	0.00920 (5)	0.613 (11)
Co3	0.14535 (2)	0.125674 (19)	−0.43994 (2)	0.00920 (5)	0.387 (11)
Co1	0.51531 (2)	0.129584 (19)	0.27594 (2)	0.00646 (4)	
Co2	0.11526 (2)	0.133641 (19)	0.03006 (2)	0.00808 (4)	
P1	0.38414 (4)	0.16385 (4)	−0.13938 (4)	0.00589 (6)	
P2	0.20915 (4)	−0.08141 (3)	−0.67010 (4)	0.00519 (6)	
O1	0.57091 (13)	0.22680 (11)	−0.09698 (12)	0.01030 (17)	
O2	0.36035 (14)	0.13059 (11)	0.01540 (12)	0.00958 (16)	
O3	0.35326 (13)	0.03970 (11)	−0.25406 (12)	0.00944 (16)	
O4	0.22753 (13)	0.25872 (11)	−0.25036 (12)	0.00952 (16)	
O5	0.08457 (14)	−0.02059 (11)	−0.59509 (12)	0.01044 (17)	
O6	0.08992 (13)	−0.18315 (10)	−0.80105 (11)	0.00813 (15)	
O7	0.37173 (13)	−0.15475 (11)	−0.53900 (12)	0.00950 (16)	
O8	0.27312 (13)	0.03376 (10)	−0.74946 (12)	0.00871 (16)	
O9	−0.10961 (14)	0.08498 (12)	0.07202 (12)	0.01199 (18)	
H9A	−0.2021	0.1199	−0.0001	0.018*	
H9B	−0.1031	0.0899	0.1749	0.018*	

### Atomic displacement parameters (Å^2^)

**Table:**

	*U*^11^	*U*^22^	*U*^33^	*U*^12^	*U*^13^	*U*^23^
Cu3	0.01172 (8)	0.00784 (8)	0.00548 (7)	0.00309 (5)	0.00105 (6)	−0.00054 (5)
Co3	0.01172 (8)	0.00784 (8)	0.00548 (7)	0.00309 (5)	0.00105 (6)	−0.00054 (5)
Co1	0.00605 (7)	0.00578 (8)	0.00694 (7)	0.00026 (5)	0.00208 (5)	−0.00043 (5)
Co2	0.00620 (7)	0.00832 (8)	0.00848 (7)	−0.00025 (5)	0.00182 (6)	0.00165 (6)
P1	0.00590 (11)	0.00584 (13)	0.00529 (12)	0.00016 (10)	0.00170 (9)	−0.00011 (10)
P2	0.00508 (11)	0.00526 (13)	0.00475 (11)	−0.00017 (9)	0.00156 (9)	0.00037 (9)
O1	0.0077 (4)	0.0124 (5)	0.0107 (4)	−0.0029 (3)	0.0037 (3)	−0.0035 (3)
O2	0.0093 (4)	0.0125 (4)	0.0068 (4)	−0.0004 (3)	0.0033 (3)	0.0012 (3)
O3	0.0091 (4)	0.0081 (4)	0.0081 (4)	0.0015 (3)	0.0006 (3)	−0.0024 (3)
O4	0.0093 (4)	0.0095 (4)	0.0086 (4)	0.0038 (3)	0.0026 (3)	0.0017 (3)
O5	0.0102 (4)	0.0120 (4)	0.0115 (4)	−0.0003 (3)	0.0069 (3)	−0.0029 (3)
O6	0.0088 (4)	0.0067 (4)	0.0067 (3)	−0.0016 (3)	0.0011 (3)	−0.0004 (3)
O7	0.0087 (4)	0.0090 (4)	0.0076 (4)	0.0012 (3)	0.0002 (3)	0.0017 (3)
O8	0.0082 (3)	0.0081 (4)	0.0092 (4)	−0.0017 (3)	0.0029 (3)	0.0024 (3)
O9	0.0104 (4)	0.0165 (5)	0.0094 (4)	0.0019 (3)	0.0045 (3)	0.0015 (3)

### Geometric parameters (Å, °)

**Table:**

Cu3—O5	1.9236 (11)	P1—O4	1.5558 (11)
Cu3—O1^i^	1.9411 (11)	P2—O7	1.5343 (11)
Cu3—O3	2.0026 (11)	P2—O8	1.5402 (11)
Cu3—O4	2.0347 (12)	P2—O6	1.5427 (11)
Cu3—O5^ii^	2.2614 (11)	P2—O5	1.5496 (10)
Co1—O3^iii^	2.0274 (11)	O1—Co3^vi^	1.9411 (11)
Co1—O6^iv^	2.0791 (11)	O1—Cu3^vi^	1.9411 (11)
Co1—O8^v^	2.0976 (11)	O3—Co1^iii^	2.0274 (11)
Co1—O4^vi^	2.1321 (11)	O4—Co1^i^	2.1320 (11)
Co1—O2	2.1588 (12)	O5—Co3^ii^	2.2613 (11)
Co1—O7^iii^	2.1779 (12)	O5—Cu3^ii^	2.2613 (11)
Co2—O2	2.0407 (11)	O6—Co1^viii^	2.0790 (11)
Co2—O9	2.0625 (11)	O6—Co2^ii^	2.1010 (11)
Co2—O7^iv^	2.0818 (13)	O7—Co2^viii^	2.0818 (12)
Co2—O6^ii^	2.1010 (11)	O7—Co1^iii^	2.1779 (12)
Co2—O8^v^	2.1088 (11)	O8—Co1^ix^	2.0976 (10)
Co2—O9^vii^	2.3310 (13)	O8—Co2^ix^	2.1087 (11)
P1—O2	1.5254 (11)	O9—Co2^vii^	2.3310 (13)
P1—O1	1.5257 (11)	O9—H9A	0.8342
P1—O3	1.5525 (11)	O9—H9B	0.9111
			
O5—Cu3—O1^i^	96.74 (5)	O9—Co2—O7^iv^	104.98 (4)
O5—Cu3—O3	99.61 (5)	O2—Co2—O6^ii^	109.20 (4)
O1^i^—Cu3—O3	145.59 (4)	O9—Co2—O6^ii^	80.81 (4)
O5—Cu3—O4	171.47 (4)	O7^iv^—Co2—O6^ii^	79.25 (4)
O1^i^—Cu3—O4	91.68 (5)	O2—Co2—O8^v^	80.38 (4)
O3—Cu3—O4	72.46 (4)	O9—Co2—O8^v^	87.19 (4)
O5—Cu3—O5^ii^	77.63 (4)	O7^iv^—Co2—O8^v^	115.29 (4)
O1^i^—Cu3—O5^ii^	114.90 (4)	O6^ii^—Co2—O8^v^	163.32 (4)
O3—Cu3—O5^ii^	98.14 (5)	O2—Co2—O9^vii^	79.64 (4)
O4—Cu3—O5^ii^	100.04 (4)	O9—Co2—O9^vii^	89.19 (4)
O3^iii^—Co1—O6^iv^	172.66 (4)	O7^iv^—Co2—O9^vii^	158.15 (4)
O3^iii^—Co1—O8^v^	97.15 (5)	O6^ii^—Co2—O9^vii^	86.90 (4)
O6^iv^—Co1—O8^v^	90.19 (4)	O8^v^—Co2—O9^vii^	81.36 (4)
O3^iii^—Co1—O4^vi^	86.13 (5)	O2—P1—O1	110.26 (6)
O6^iv^—Co1—O4^vi^	86.65 (4)	O2—P1—O3	113.44 (6)
O8^v^—Co1—O4^vi^	167.68 (4)	O1—P1—O3	110.69 (6)
O3^iii^—Co1—O2	89.25 (4)	O2—P1—O4	109.89 (6)
O6^iv^—Co1—O2	92.14 (4)	O1—P1—O4	111.95 (6)
O8^v^—Co1—O2	77.97 (4)	O3—P1—O4	100.30 (6)
O4^vi^—Co1—O2	90.24 (4)	O7—P2—O8	111.04 (6)
O3^iii^—Co1—O7^iii^	101.61 (4)	O7—P2—O6	110.42 (6)
O6^iv^—Co1—O7^iii^	77.57 (4)	O8—P2—O6	110.02 (6)
O8^v^—Co1—O7^iii^	96.78 (4)	O7—P2—O5	110.30 (6)
O4^vi^—Co1—O7^iii^	94.16 (4)	O8—P2—O5	109.04 (6)
O2—Co1—O7^iii^	168.52 (4)	O6—P2—O5	105.87 (6)
O2—Co2—O9	164.36 (4)	H9A—O9—H9B	115.3
O2—Co2—O7^iv^	89.00 (4)		

Symmetry codes: (i) *x*−1/2, −*y*+1/2, *z*−1/2; (ii) −*x*, −*y*, −*z*−1; (iii) −*x*+1, −*y*, −*z*; (iv) −*x*+1/2, *y*+1/2, −*z*−1/2; (v) *x*, *y*, *z*+1; (vi) *x*+1/2, −*y*+1/2, *z*+1/2; (vii) −*x*, −*y*, −*z*; (viii) −*x*+1/2, *y*−1/2, −*z*−1/2; (ix) *x*, *y*, *z*−1.

### Hydrogen-bond geometry (Å, °)

**Table:**

*D*—H···*A*	*D*—H	H···*A*	*D*···*A*	*D*—H···*A*
O9—H9A···O1i	0.83	1.97	2.768 (2)	159
O9—H9B···O5^v^	0.92	2.27	2.942 (2)	130
O9—H9B···O4^x^	0.92	2.30	2.905 (2)	123

Symmetry codes: i; (v) *x*, *y*, *z*+1; (x) *x*−1/2, −*y*+1/2, *z*+1/2.
